# Shape-Memory–Assisted Self-Healing and Real-Time Acidic Environment Detection in Multifunctional Electrospun Fibers

**DOI:** 10.1021/acsami.5c24793

**Published:** 2026-01-30

**Authors:** Huan-Ru Chen, Yi-Fan Chen, Tse-Yu Lo, Chien-Lin Chen, Kai-Jie Chang, Kuan-Hsun Tseng, Jhih-Hao Ho, Jiun-Tai Chen

**Affiliations:** † Department of Applied Chemistry, 34914 National Yang Ming Chiao Tung University, 300093 Hsinchu, Taiwan; ‡ Center for Emergent Functional Matter Science, 34914 National Yang Ming Chiao Tung University, 300093 Hsinchu, Taiwan

**Keywords:** shape-memory polymers, self-healing fibers, electrospinning, acidochromic materials, smart textiles

## Abstract

Smart fiber systems that integrate self-healing and environmental responsiveness are emerging as promising candidates for wearable electronics, protective clothing, and adaptive textiles. Here, we report a multifunctional electrospun fiber platform combining shape-memory–assisted self-healing with acid-triggered chromism. The fibers are fabricated from thermoplastic polyurethane (TPU)/poly­(ε-caprolactone) (PCL) blends doped with the acid-sensitive dye ODB-2. Distinct thermal transitions of TPU and PCL enable programmable deformation and recovery, whereby fractured fibers self-heal through thermally activated interfacial diffusion. Meanwhile, ODB-2 undergoes a reversible structural change upon protonation, producing a visible color contrast that functions as an acid-responsive optical signal. Systematic characterization of chemical structure, morphology, and functional performance reveals a clear compositional dependence. TPU-rich blends yield uniform fiber morphologies, stable chromic reversibility over multiple acid–base cycles, and self-healing efficiencies up to 95%. In contrast, PCL-rich compositions exhibit larger fiber diameters, reduced chromic response, and incomplete mechanical recovery. These results demonstrate how polymer composition governs both structural features and functional outcomes. By integrating shape-memory, self-healing, and chromic responsiveness into a single platform, this work establishes a versatile design strategy for smart polymer fibers capable of damage repair and real-time environmental monitoring. The approach offers broad opportunities for developing next-generation wearable electronics, intelligent textiles, and adaptive membranes for use in corrosive or dynamically fluctuating environments.

## Graphical abstract

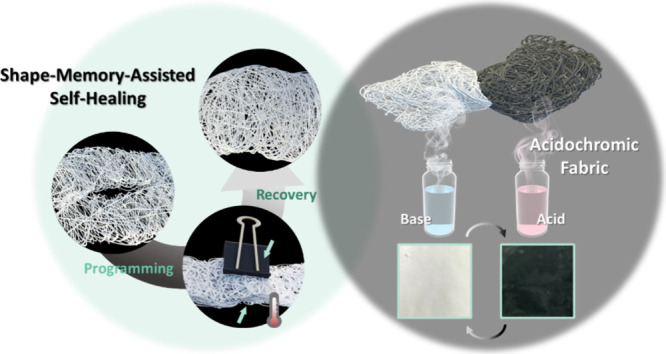

## Introduction

Smart materials that integrate self-healing and environmental responsiveness are gaining increasing attention for applications in wearable electronics,^1,2^ soft robotics,^3,4^ and adaptive textiles.^5–7^ Among these, shape-memory polymers (SMPs) stand out as they can recover their original form upon external stimuli such as temperature, particularly when constituted by components with distinct thermal transitions and physical cross-links.^8,9^ Poly­(ε-caprolactone) (PCL), a semicrystalline polyester with a well-defined melting transition, is a widely used shape-memory polymer.^10,11^ Its crystalline domains act as reversible “switching segments” that fix temporary shapes at low temperature and enable recovery upon heating above the melting point.^12,13^ In the literature, PCL has been combined with various polymers such as polyurethanes,^12,14^ poly­(lactic acid),^15,16^ and poly­(ethylene glycol)^17,18^ to form SMP systems, where the complementary thermal or mechanical properties enhance shape fixation, recovery speed, and overall durability. Thermoplastic polyurethane (TPU), a segmented elastomer with microphase-separated soft and hard domains, serves as an elastic matrix providing mechanical robustness and reversible physical cross-links through hydrogen bonding.^19–21^ Owing to its high elasticity and intrinsic self-healing capability, TPU is frequently combined with PCL to construct shape-memory systems that integrate efficient shape recovery with mechanical durability and damage tolerance.^22,23^ Notably, electrospun TPU/PCL fiber systems have been reported to exhibit favorable mechanical performance and structural integrity in fibrous architectures, highlighting their suitability for shape-memory and functional fiber applications.^24,25^

In parallel, electrospinning has emerged as a versatile method for generating fibrous architectures with high surface area and tunable morphology.^26,27^ Electrospun fibers of different polymers and their composites are extensively utilized in biomedical dressings, tissue scaffolds, and responsive materials owing to their porosity and mechanical adaptability.^28–32^ Nevertheless, their inherent softness makes them prone to mechanical damage, which can lead to premature failure, material waste, and inconvenience.^33–35^ Self-healing polymers offer a promising strategy to address this limitation by enabling the restoration of mechanical integrity after damage through intrinsic repair mechanisms, such as hydrogen bonds, dynamic covalent chemistry, or phase softening, thereby allowing recovery from damage under mild stimuli.^36–38^ In particular, intrinsically self-healing systems based on reversible covalent bonds have attracted significant interest, as thermally activated bond dissociation and reformation can effectively restore network connectivity without the need for external healing agents.^39,40^

Incorporating self-healing capability into SMP systems further enables shape-memory-assisted healing, whereby thermal activation of switching segments not only triggers macroscopic shape recovery but also enhances chain mobility and interfacial diffusion, thereby accelerating healing and improving mechanical recovery.^41–44^

Recent studies have demonstrated that such systems can achieve remarkable levels of mechanical recovery when the interplay between switching and cross-linking components is carefully optimized.^45,46^ Most self-healing SMP-based fibers, however, focus solely on mechanical recovery. Incorporating visual responsiveness, such as chromic signaling upon environmental stimuli (e.g., pH, gases),^47–50^ enables real-time monitoring of external chemical exposure, providing complementary information to the self-healing functionality.^51–53^ Here, we present a multifunctional fiber system that integrates shape-memory–assisted self-healing with acid-triggered chromism. Electrospun fibers are fabricated from thermoplastic polyurethane (TPU)/poly­(ε-caprolactone) (PCL) blends doped with the acid-sensitive dye ODB-2. Owing to the distinct thermal transitions of PCL and TPU, the fibers exhibit programmable deformation and recovery, enabling fractured segments to self-heal through thermally activated interfacial adhesion that effectively welds fibers together at specific temperatures. Meanwhile, ODB-2 undergoes a reversible color change upon protonation, providing visible acid-responsive signaling. A systematic investigation of chemical structure, morphology, chromic response, and healing performance reveals the strong coupling between polymer composition and multifunctional behavior. By uniting self-healing, shape-memory, and environmental sensing in a single platform, these fibers offer promising opportunities for wearable electronics, protective clothing, and adaptive textiles in hazardous environments with fluctuating acidity or corrosive gases.

## Experimental Section

### Materials

Polycaprolactone (PCL, *M*_n_ = 80,000 g mol^–1^) was sourced from Sigma-Aldrich. Commercial thermoplastic polyurethane (TPU, 1685A-E2; Shore A hardness of 83; elongation at break of 850%; specific gravity of 1.2) was provided by Great Eastern Resin Industry Co., Ltd. 2-Anilino-6-dibutylamino-3-methylfluoran (ODB-2) was obtained from Combi-Blocks. Dimethylformamide (DMF), tetrahydrofuran (THF), and ethanol (>99.0%) were received from Thermo Scientific, Macron Fine Materials, and Honeywell, respectively. Trifluoroacetic acid (TFA) and ammonium hydroxide (28–30%) were supplied by Thermo Scientific and J.T. Baker, respectively. All the chemicals were used without further purification.

### Preparation of Acid-Responsive Self-Healing Polymer Solution

TPU and PCL were weighed at ratios of 8:2, 6:4, 4:6, and 2:8 (TPU:PCL) to a total mass of 1.5 g. ODB-2 powder (60 mg) was then added, and all solutes were placed into a 10 mL sample vial. A mixed solvent consisting of 2.7 g of DMF and 1.8 g of THF (molar ratio = 1.5:1) was used to dissolve the solids. After adding a magnetic stir bar, the vial was sealed with parafilm and stirred at 40 °C and 150 rpm on an electromagnetic stirrer for 10 h, yielding a 25 wt % acid-responsive self-healing solution.

### Electrospinning of Acid-Responsive Self-Healing Fibers

The homogeneous polymer solution (8:2, 6:4, 4:6, and 2:8 (TPU:PCL)) was processed into fibers using horizontal electrospinning under the following conditions. A 5 mL polypropylene syringe was loaded with 3 mL of the polymer solution; the remaining solution was added in two portions during spinning to minimize solvent evaporation. A stainless-steel needle (inner diameter 0.41 mm) was attached to the syringe and mounted on a microinfusion pump (KD Scientific), with the flow rate set to 1.5 mL h^–1^. The rotating drum collector (Falco; diameter 10 cm, covered with baking paper) was positioned 15 cm horizontally from the needle tip and rotated at 700 rpm. A power supply (SMICO) with a high voltage of 20 kV was applied by connecting the positive electrode to the needle, while the collector was grounded, thereby generating an electric field that drew fibers toward the collector. After electrospinning all the solution, the fibers were removed together with the baking paper and stored in a dry, room-temperature environment.

### Mechanical Strength of Acid-Responsive Self-Healing Fabrics

The electrospinning process used to fabricate the acid-responsive self-healing fabrics was adapted from our previous work with minor modifications.^30^ Mechanical behavior of the hydrochromic fabrics was assessed using a tensile testing system (Shimadzu EZ Test) equipped with a 500 N load cell under uniaxial extension. TPU/PCL hydrochromic fabrics were cut into strips measuring 1 × 3 cm^2^ and mounted onto the grips of a tensile testing machine. Uniaxial stretching was performed under displacement control at a rate of 6 mm min^–1^ to determine the elongation at break. For cyclic loading tests, the displacement rate was adjusted to 8 mm min^–1^, with each cycle stretched to a fixed elongation length of 16 mm, followed by relaxation. A total of 20 loading–unloading cycles were carried out. The cyclic loading–unloading tests (20 cycles) were performed after a single self-healing treatment to evaluate the mechanical durability of the healed interface under repeated deformation.

### Structure Characterizations and Analyses

Thermal degradation temperature (*T*_d_, 5 wt % weight loss) of ODB-2 powders, as well as TPU and TPU/PCL hydrochromic fabrics, was measured using a thermogravimetric analyzer (TGA, TA 55, TA Instruments). Each specimen was heated to 700 °C at 10 °C min^–1^ under a nitrogen atmosphere. The glass transition (*T*_g_) and melting points (*T*_m_) of the same materials were characterized with a differential scanning calorimeter (DSC, TA Q200, TA Instruments) through two heating–cooling cycles between 0 and 180 °C at 10 °C min^–1^ under N_2_. Surface morphologies of TPU and TPU/PCL fibers were visualized by field-emission scanning electron microscopy (SEM, JEOL JSM-7401F) operated at 5 kV. Fiber diameters (≈30 per sample) were determined from SEM images using ImageJ. X-ray diffraction (XRD) measurements of the fabrics were performed at the 13A BioSWAXS beamline of the Taiwan Photon Source (TPS) at the National Synchrotron Radiation Research Center (NSRRC). The color-responsive performance was evaluated with a UV–vis spectrophotometer (Hitachi U4100) in reflective mode.

## Results and Discussion

To realize multifunctional fibers capable of both self-healing and environmental responsiveness, we design an electrospun composite system combining shape-memory polymer blends and an acidochromic dye (Figure 1). The fibrous matrix consists of poly­(ε-caprolactone) (PCL) and thermoplastic polyurethane (TPU), two polymers with distinct thermal transition profiles that endow the material with shape-memory behavior. PCL provides crystalline domains with a melting point around 60 °C, which soften upon heating to enable chain mobility, while the hydrogen-bonded hard segments of TPU act as physical cross-links that maintain network integrity. This combination allows the fibers to be mechanically programmed, deformed, and subsequently recovered upon thermal activation.

**Figure 1:**
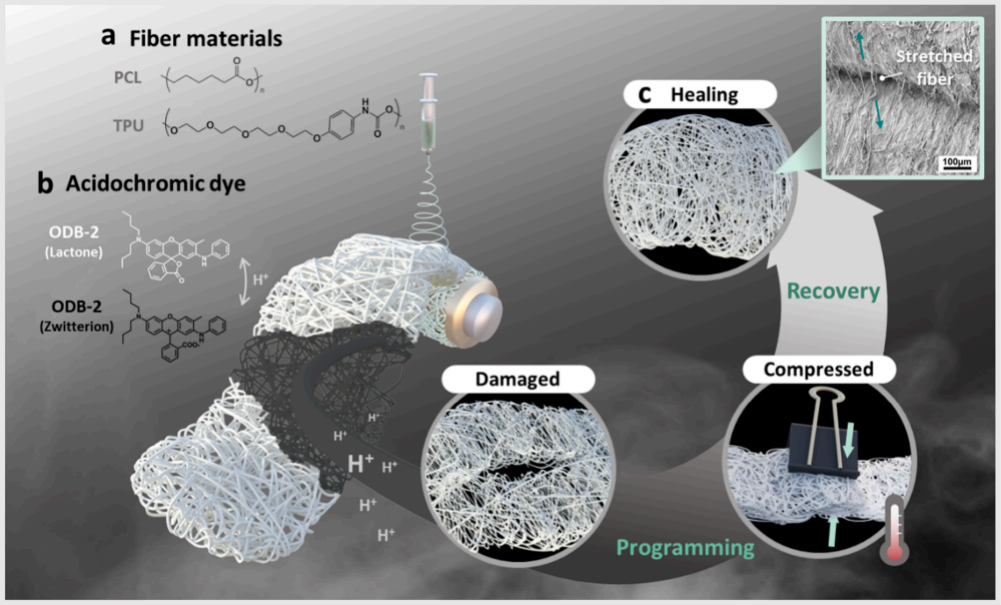
Conceptual illustration of acidichromic self-healing fibers. (a) Fiber materials composed of poly­(ε-caprolactone) (PCL) and thermoplastic polyurethane (TPU). (b) Acidichromic indicator ODB-2, which undergoes a reversible structural transformation between the lactone and zwitterionic states in response to protons (H^+^). (c) Schematic of the shape-memory-assisted healing cycle. Electrospun fibers are first compressed and programmed under thermal stimulus, then mechanically damaged, and subsequently restored to their original fibrous morphology upon recovery.

To integrate environmental responsiveness, we incorporate the acidochromic dye ODB-2 into the fibers. ODB-2 undergoes a reversible transition from its colorless lactone form to a zwitterionic state upon protonation, leading to a pronounced optical contrast. This feature enables the fibers not only to self-heal mechanically but also to visually report local proton concentrations. The interplay between shape-memory programming and chromic response underpins the self-healing mechanism. As illustrated in Figure 1c, the fibers are first compressed under mild heating to induce temporary fixation of the deformed structure. Upon mechanical damage, the fibrous network becomes disrupted; however, reheating above the PCL melting point reactivates chain mobility, enabling interfacial diffusion and re-entanglement of polymer chains across fractured regions. After removal of the applied pressure and subsequent cooling to room temperature, shape recovery occurs, during which polymer chains across the interface re-entangle and effectively weld the damaged regions together. This thermally activated recovery process distinguishes the self-healing behavior from simple pressure-induced adhesion.

This recovery process restores the fibrous morphology and mechanical integrity. The inset micrograph confirms that the healed fibers maintain continuous structures even under tensile stretching, demonstrating effective repair. Overall, Figure 1 establishes the conceptual framework of acidichromic self-healing fibers: TPU/PCL blends provide the shape-memory-driven recovery mechanism, while ODB-2 introduces an acid-triggered optical signal, together offering a dual-function platform for intelligent and adaptive textile applications.

The chemical structures and composition of the TPU/PCL blends are characterized, as shown in Figure 2a. PCL provides semicrystalline domains, while TPU contains aromatic and urethane groups that serve as hydrogen-bonded hard segments. Their combination is expected to produce a dual-phase morphology that underlies the shape-memory effect. Fourier-transform infrared spectroscopy (FTIR) confirms the successful blending of TPU and PCL at different weight ratios (Figure 2b). Characteristic peaks corresponding to N–H stretching, C=O stretching, and aromatic vibrations are observed, with their relative intensities varying systematically with polymer composition. This result demonstrates that the molecular features of both components are retained in the blends. Thermal transitions are evaluated by differential scanning calorimetry (DSC). As shown in Figure 2c, pristine PCL exhibits a sharp melting peak at 54.24 °C, while pristine TPU shows a glass transition at −36.4 °C. Detailed DSC curves of pure TPU and PCL are presented in Figure S1. The TPU/PCL blends maintain both features, indicating that each polymer contributes distinct thermal domains. The coexistence of soft and hard segments provides the thermal triggers necessary for programming and recovery during the self-healing process.

**Figure 2:**
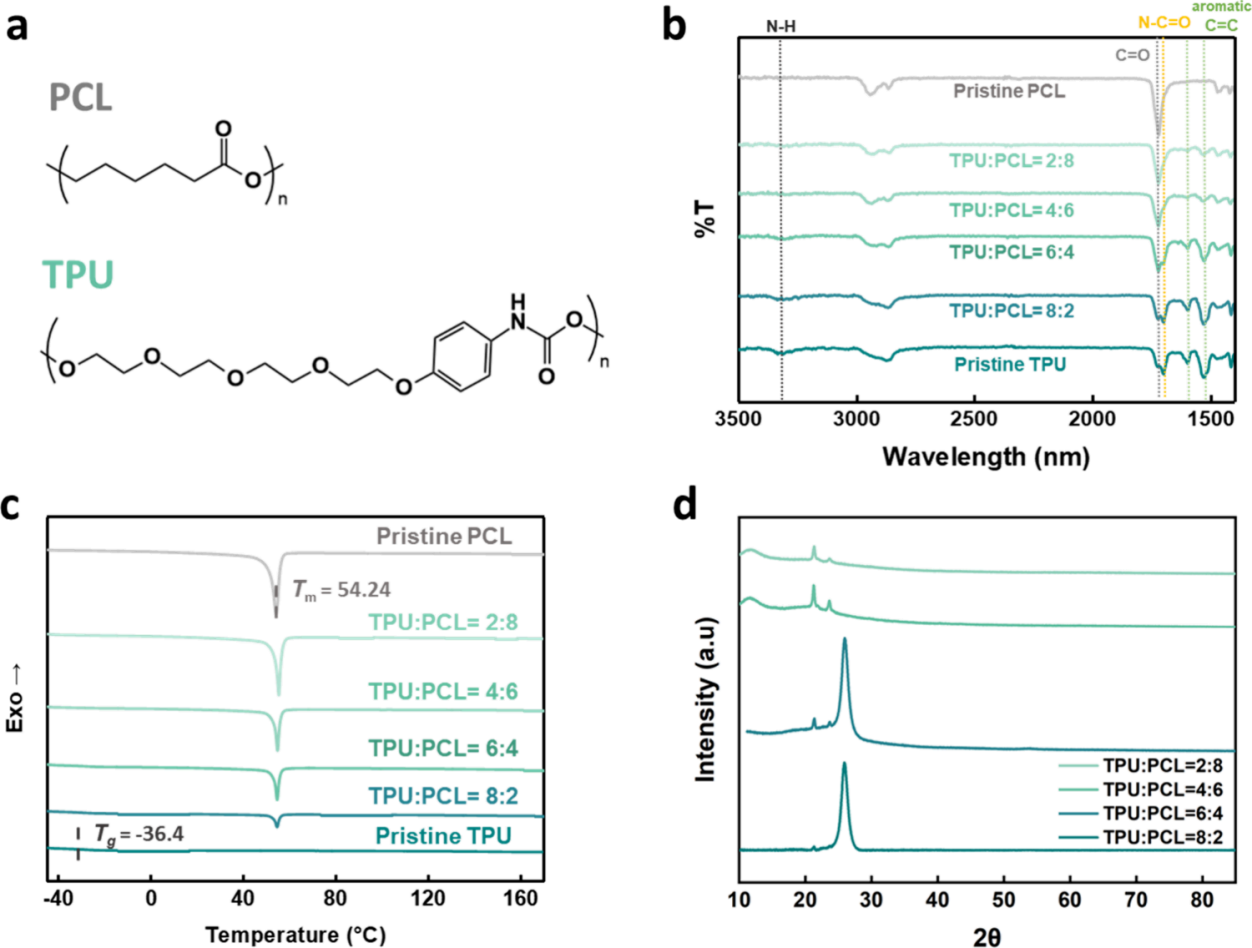
Structural and thermal characterization of TPU/PCL blends. (a) Chemical structures of PCL and TPU. (b) FTIR spectra of pristine PCL, pristine TPU, and TPU/PCL blends with varying weight ratios, showing characteristic absorption peaks corresponding to N–H, C=O, and aromatic groups. (c) DSC curves of pristine PCL, pristine TPU, and TPU/PCL blends. (d) XRD patterns of TPU/PCL blends prepared from DMF/THF solutions, indicating crystalline features and structural evolution with different TPU:PCL ratios.

X-ray diffraction (XRD) further reveals that the crystalline order of PCL is modulated by the TPU content (Figure 2d). Increasing TPU fraction leads to broader diffraction peaks, reflecting reduced crystallinity and enhanced chain mixing. These findings confirm that the TPU/PCL blends form tunable semicrystalline networks in which crystalline PCL domains enable temporary shape fixation and TPU segments preserve structural elasticity, together establishing the structural foundation for subsequent self-healing behavior.

We investigate the morphological evolution of TPU/PCL electrospun fibers as a function of polymer composition (Figure 3). SEM observations (Figure 3b–e) show that all blends form continuous fibrous mats, yet differences in diameter and uniformity emerge. As summarized in Figure 3a, the average fiber diameter increases progressively with higher PCL content, highlighting the role of polymer solubility and phase compatibility in determining electrospinning behavior. The diameter distributions further illustrate this trend (Figure 3f–i). TPU-rich fibers (8:2 and 6:4) exhibit narrow Gaussian distributions centered around 3 μm, reflecting stable jet formation and uniform chain entanglement during spinning. In contrast, blends with higher PCL content (4:6 and 2:8) produce broader or even bimodal distributions. Particularly at TPU:PCL= 2:8, the fibers show branched morphologies and diameters exceeding 8 μm, indicating that phase separation becomes more pronounced when PCL dominates the system.

**Figure 3:**
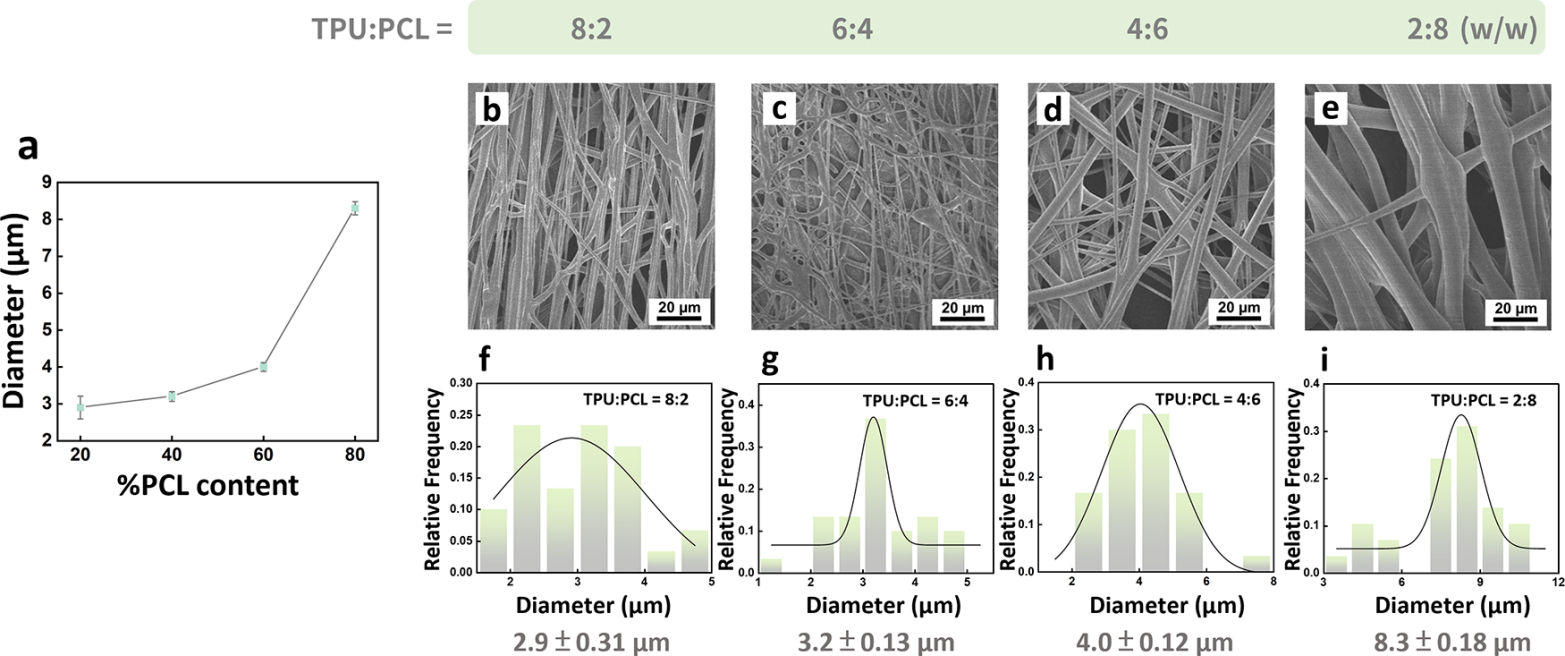
Morphology and diameter distribution of TPU/PCL electrospun fibers with varying PCL contents. (a) Average fiber diameter as a function of PCL content, showing an increase in diameter at higher PCL ratios. (b–e) SEM images of electrospun fibers with TPU:PCL ratios of (b) 8:2, (c) 6:4, (d) 4:6, and (e) 2:8 (w/w). (f–i) Fiber diameter distributions corresponding to (b–e), fitted with Gaussian curves and showing average diameters.

This compositional effect originates from differences in polymer–solvent affinity. Figure S2 compares the fiber morphologies obtained from mixed DMF/THF and single-solvent DMF systems, demonstrating that solvent volatility plays a critical role in achieving uniform, well-separated electrospun fibers. TPU dissolves readily in the DMF/THF mixture, producing homogeneous solutions that facilitate uniform electrospinning. PCL, however, is less soluble in THF, leading to partial aggregation during jet stretching. Under the high-voltage field, these aggregates destabilize the electrospinning process and generate heterogeneous fiber morphologies. These findings establish a correlation between blend composition and fiber structure. TPU-rich blends favor uniform fiber formation, while PCL-rich systems promote thicker and branched morphologies that later influence both the chromic response and the self-healing behavior of the fibers. Figure S3 presents SEM and EDS nitrogen mapping analyses of TPU/PCL fibers and films with varying compositions, demonstrating that electrospinning suppresses macroscopic phase separation and promotes TPU surface enrichment compared to corresponding drop-cast films.

We next evaluate the acidochromic response of the TPU/PCL electrospun fibers (Figure 4a). Upon exposure to trifluoroacetic acid (TFA) vapor, the fibers exhibit a distinct and rapid color change from off-white to deep green, which corresponds to the protonation and ring-opening transition of the incorporated ODB-2 dye. The optical signal is readily visible by eye and is further quantified by UV–vis reflectance spectroscopy. The reflectance difference at 583 nm provides a sensitive measure of chromic response in different TPU/PCL ratios (Figure 4b). TPU-rich fibers (TPU:PCL = 8:2) display the largest reflectance drop, while PCL-rich fibers show weaker color contrast. This difference arises because TPU segments contain aromatic and urethane groups that promote stronger dye–polymer interactions, stabilizing ODB-2 in its closed form and enhancing its optical switching upon protonation. Spectral comparisons before and after acid exposure confirm this behavior across different blend ratios (Figure 4c–f). TPU-rich compositions retain high reflectance in the pristine state and undergo pronounced spectral shifts under TFA vapor, while increasing PCL content leads to reduced switching amplitude.

**Figure 4:**
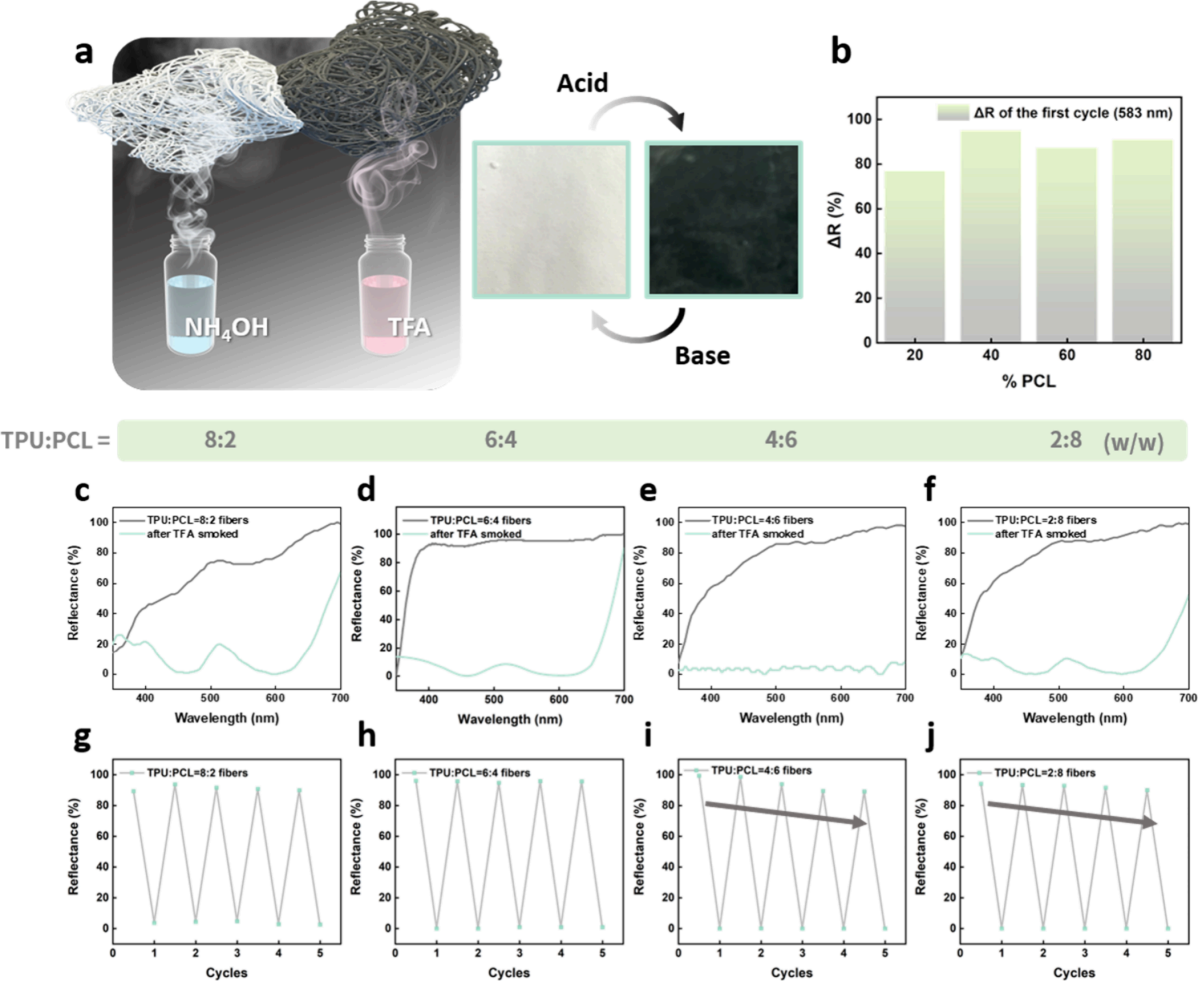
Acidichromic response and reversibility of TPU/PCL electrospun fibers. (a) Graphical illustration and optical images of fibers before (white) and after (dark green) TFA exposure, demonstrating the visible acidochromic effect. (b) Reflectance change (Δ*R*) at 583 nm during the first acid exposure cycle as a function of PCL content. (c–f) UV–vis reflectance spectra of TPU/PCL fibers with ratios of (c) 8:2, (d) 6:4, (e) 4:6, and (f) 2:8 (w/w), before and after exposure to TFA vapor, showing a pronounced decrease in reflectance upon protonation. (g–j) Cyclic reversibility tests of fibers with TPU:PCL ratios of (g) 8:2, (h) 6:4, (i) 4:6, and (j) 2:8 over five acid–base cycles, showing stable color switching in TPU-rich fibers and reduced recovery in PCL-rich compositions.

We further test reversibility by alternating acid and base vapors over multiple cycles (Figure 4g–j). Fibers with higher TPU fractions show stable and repeatable switching over at least five cycles, whereas those with higher PCL content gradually lose recovery, consistent with weaker dye–polymer stacking and fewer stabilizing hydrogen bonds. This trend highlights that polymer composition not only dictates fiber morphology but also governs the robustness of acid-responsive coloration. It should be noted that ODB-2 is a fluorane-based acidochromic dye, and potential skin irritation associated with small-molecule dyes in their free molecular form cannot be excluded. In the present system, ODB-2 is physically immobilized within the TPU/PCL fiber matrix, which is expected to limit direct skin contact and dye leaching; nevertheless, comprehensive biocompatibility and skin irritation evaluations will be required prior to practical wearable applications.

We examine the self-healing performance of TPU/PCL electrospun fibers driven by their shape-memory effect (Figure 5a). This healing process follows a shape memory cycle involving contact-induced programming, thermal activation, and subsequent shape recovery.^22,23,54^ For the self-healing demonstration, electrospun fiber mats are first cut into rectangular specimens and overlapped at the fractured region. A mild external pressure is applied to ensure intimate interfacial contact between the damaged fiber mats. The overlapped samples are subsequently heated in a convection oven at 65 °C for 30 min, a temperature above the melting point of the PCL switching segments. This thermal treatment corresponds to the programming stage of the shape memory cycle, during which enhanced polymer chain mobility enables interfacial diffusion and partial interpenetration across the damaged interface. After the heating step, the external pressure is removed, and the samples are allowed to cool naturally to room temperature. During this cooling process, shape recovery occurs, restoring the original fibrous morphology and reconnecting the fractured regions through polymer chain re-entanglement. After cooling to room temperature, the healed samples recover mechanical integrity, as shown by tensile stretching without fracture (Figure 5b). Importantly, this process is not governed by pressure-induced adhesion alone, but by thermally activated shape recovery that facilitates polymer chain re-entanglement across the interface. Quantitative evaluation of healing efficiency reveals a strong dependence on polymer composition (Figure 5c). TPU:PCL = 8:2 fibers achieve the highest efficiency of ∼95%, while the value decreases to ∼80% at intermediate ratios (6:4 and 4:6) and drops sharply to ∼49% in the PCL-rich 2:8 system. This trend reflects the balance between PCL-induced softening, which facilitates chain mobility, and TPU-provided hydrogen bonding, which contributes to shape recovery and structural cohesion. Figure S4 presents amplitude-sweep and temperature-dependent rheological analyses of TPU, TPU/PCL, and PCL, revealing distinct viscoelastic softening, recovery, and modulus evolution behaviors that reflect their composition-dependent chain mobility and thermal response.

**Figure 5:**
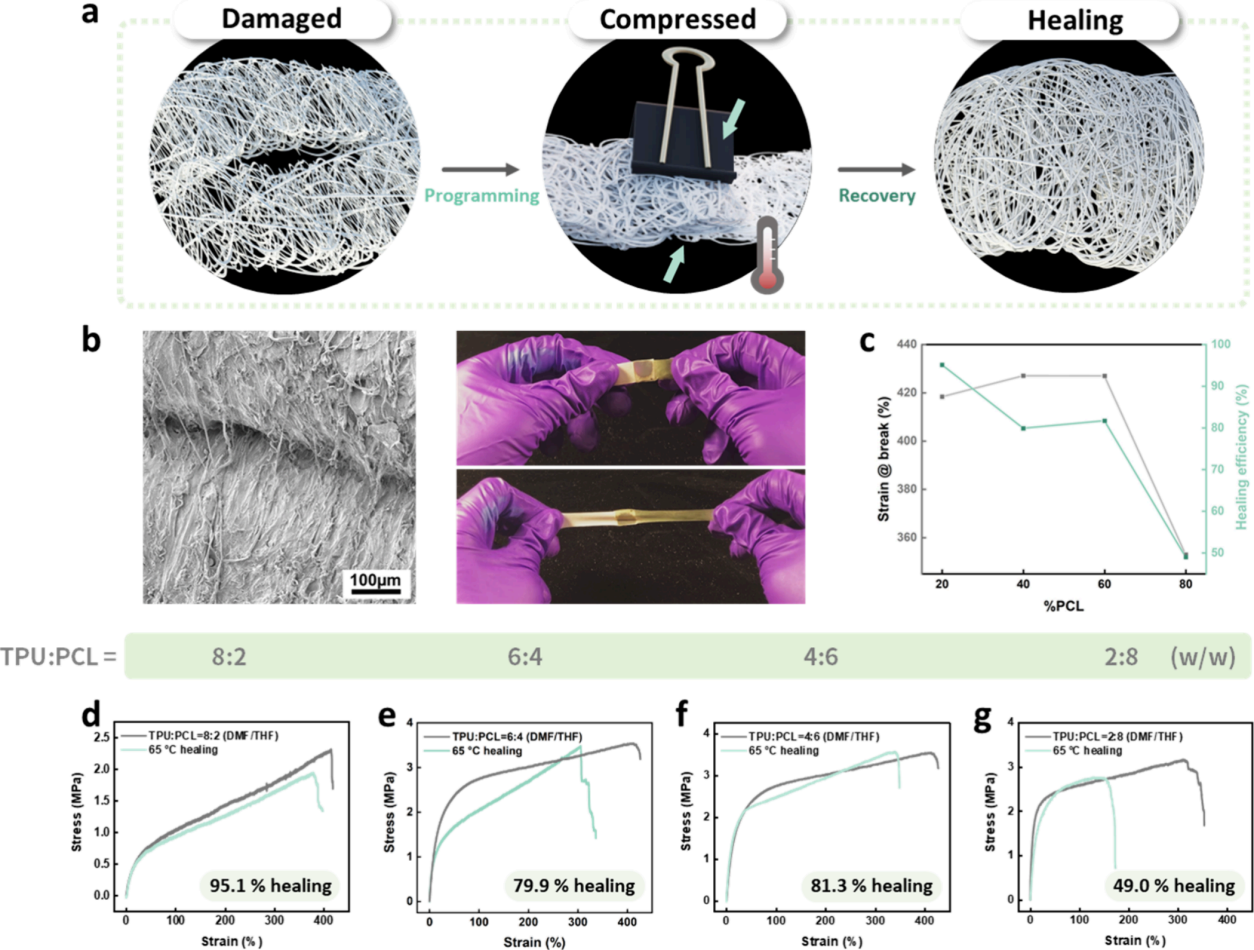
Shape-memory-assisted self-healing behavior of TPU/PCL electrospun fibers. (a) Graphical illustrations of the shape-memory-assisted self-healing. (b) Photographs and SEM images of a healed fiber strip stretched without fracture after thermal recovery. (c) Strain at break and corresponding healing efficiency as a function of PCL content, showing optimal recovery at intermediate TPU:PCL ratios. (d–g) Representative stress–strain curves of healed fibers with TPU:PCL ratios of (d) 8:2, (e) 6:4, (f) 4:6, and (g) 2:8 (w/w).

Representative stress–strain curves further illustrate these effects (Figure 5d–g). TPU-rich fibers show nearly full recovery of strain at break after healing, while PCL-rich compositions exhibit reduced tensile strength and incomplete recovery. The change in slope before fracture in healed samples suggests localized thickening at the overlapped regions, consistent with partial interdiffusion of polymer chains during the healing process. These results confirm that both thermal activation and polymer composition are critical in enabling effective shape-memory-assisted self-healing. TPU provides physical cross-linking and hydrogen bonding to drive elastic recovery, while PCL supplies the thermal trigger for chain mobility, together producing efficient repair in optimized blends. Figure S5 demonstrates that the mechanical recovery, healing efficiency, and residual deformation of TPU:PCL fibers strongly depend on the healing temperature.

The shape-memory-assisted self-healing demonstrated in this work is thermally triggered and requires external heating above the melting temperature of PCL (∼60 °C). The system is therefore not designed to autonomously heal at normal human body temperature (36–37 °C). At physiological temperatures, the fibers remain dimensionally stable without significant chain mobility or shape recovery, which is advantageous for preventing unintended deformation during wear. Future work may explore alternative switching segments with lower transition temperatures or conduct gradient temperature studies to tailor the healing behavior more closely to physiological conditions, depending on specific application requirements.

## Conclusions

In this work, we demonstrate a multifunctional electrospun fiber platform that integrates shape-memory–assisted self-healing with acid-triggered chromic signaling. By blending thermoplastic polyurethane (TPU) and poly­(ε-caprolactone) (PCL), we establish a dual-phase architecture in which crystalline PCL domains provide thermal switching and TPU segments maintain network elasticity through hydrogen bonding. The incorporation of ODB-2 further endows the fibers with visible acidochromic signaling, enabling direct optical feedback under acidic environments.

Compositional tuning governs not only fiber morphology and crystallinity but also the robustness of chromic response and the efficiency of self-healing. TPU-rich fibers exhibit uniform morphologies, stable color switching over repeated acid–base cycles, and healing efficiencies approaching 95%, whereas PCL-rich systems suffer from morphological heterogeneity, weaker acidochromism, and diminished recovery. These findings highlight the critical interplay between polymer composition, microstructure, and multifunctional behavior.

The integration of self-healing, shape-memory behavior, and visual chemical responsiveness positions this fibrous system as a promising candidate for intelligent textiles and wearable materials that require both mechanical damage tolerance and real-time environmental monitoring. Potential applications include protective clothing for acidic or corrosive environments, reusable sensing fabrics, and adaptive membranes capable of signaling damage or chemical exposure while maintaining structural integrity under dynamically fluctuating conditions. Beyond textile-based systems, the design strategy demonstrated here—combining thermally programmable polymers with reversible supramolecular interactions and chromic dyes—provides a versatile framework that can be extended to other polymer platforms, enabling the development of next-generation multifunctional materials that bridge mechanical resilience with sensory functionality for smart and responsive applications.

## Supplementary Material



## Notes

DSC curves of pristine TPU and PCL polymer films; SEM images of fibers prepared from different solvent systems; EDS analyses of polymer films with different TPU/PCL ratios; rheological response of TPU, TPU/PCL, and PCL fibers; and stress–strain curves comparing the mechanical response of healed fibers after healing at different temperatures (PDF)
