# Supplementary material for: Shape-Memory–Assisted Self-Healing and Real-Time Acidic Environment Detection in Multifunctional Electrospun Fibers
Source: ACS Appl Mater Interfaces. 2026 Jan 30;18(5):8944–52. doi: 10.1021/acsami.5c24793 (PMC12903105; doi:10.1021/acsami.5c24793)
Supplement: Supplementary file 1 [file am5c24793_si_001.pdf]

Supporting Information

Shape-Memory–Assisted Self-Healing and Real-Time Acidic Environment Detection in Multifunctional Electrospun Fibers

Huan-Ru Chen,[1#] Yi-Fan Chen,[1#] Tse-Yu Lo,[1] Chien-Lin Chen,[1] Kai-Jie Chang,[1] Kuan-Hsun Tseng,[1] Jhih-Hao Ho,[1] and Jiun-Tai Chen[1,2*]

[1]Department of Applied Chemistry, National Yang Ming Chiao Tung University, 300093 Hsinchu, Taiwan

[2]Center for Emergent Functional Matter Science, National Yang Ming Chiao Tung University, 300093 Hsinchu, Taiwan

[#]H.-R.C. and Y.-F.C. contributed equally to this work.

*To whom correspondence should be addressed. E-mail: jtchen@nycu.edu.tw

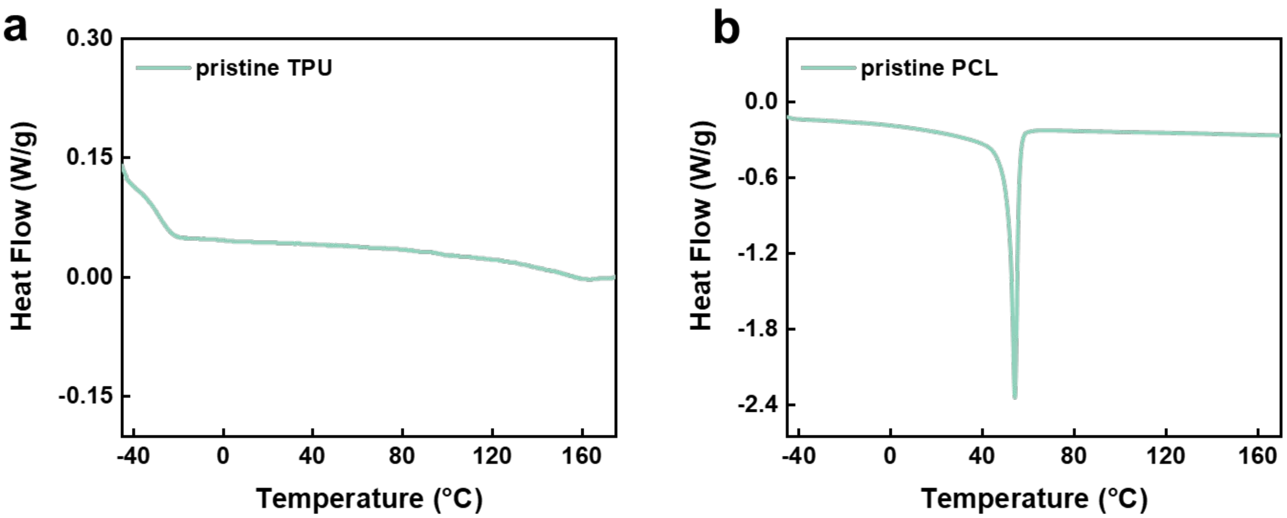

Figure S1. DSC curves of pristine (a) TPU and (b) PCL.

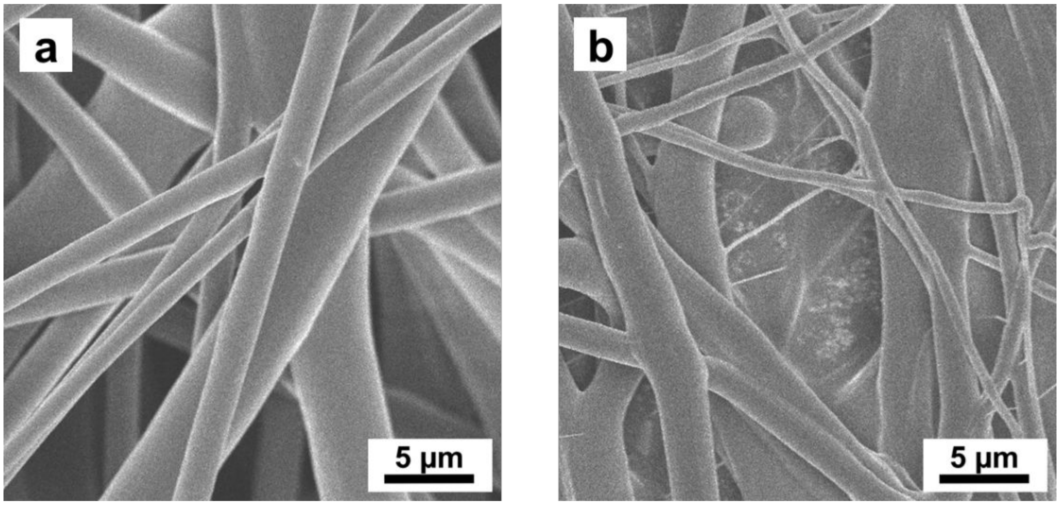

Figure S2. SEM images of fibers prepared from (a) a mixed DMF/THF solvent system and (b) DMF as a single solvent.

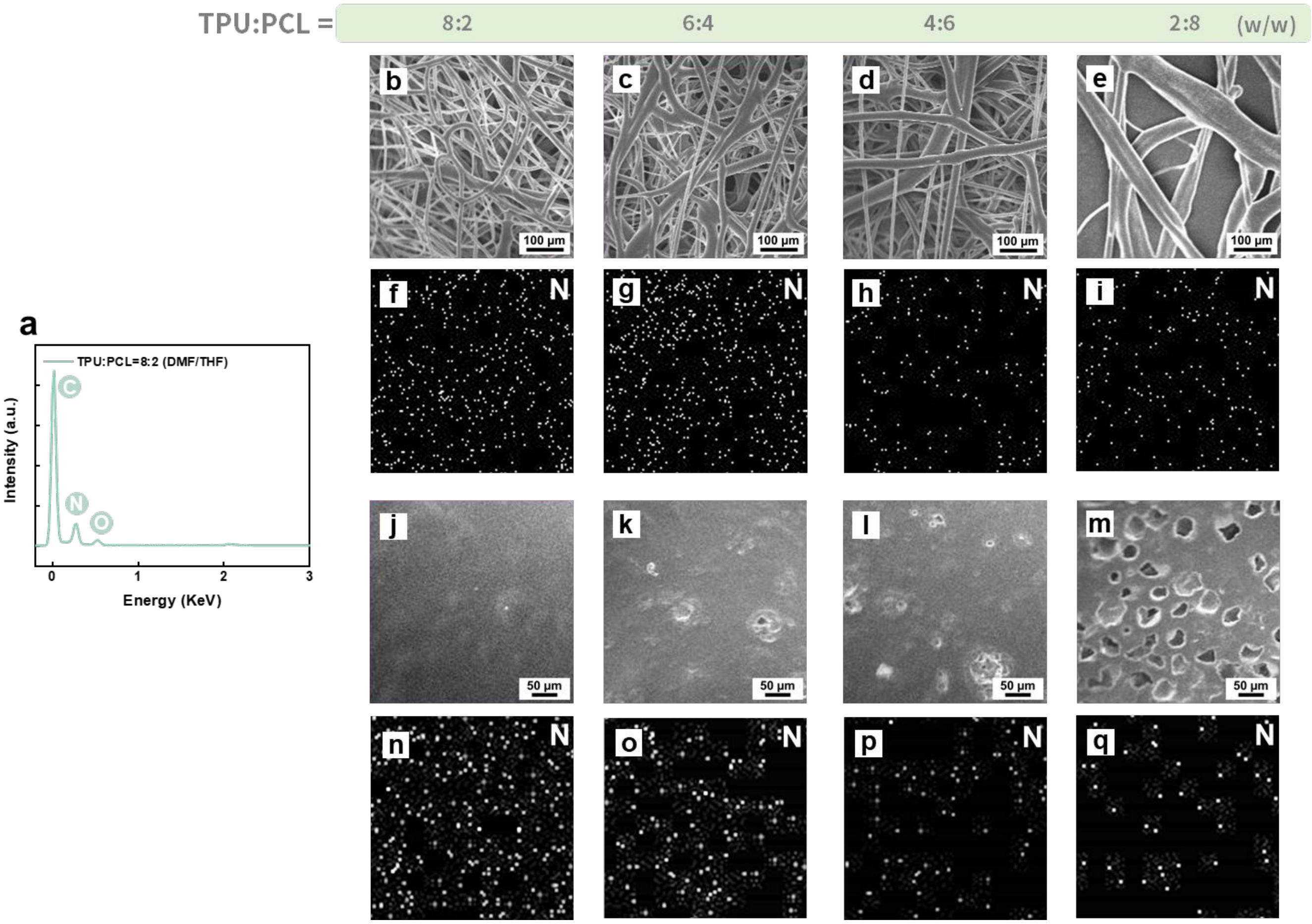

Figure S3. (a) Energy-dispersive X-ray spectroscopy (EDS) spectrum of TPU:PCL = 8:2 fibers. (b–e) SEM images of TPU/PCL fibers. (f–i) Nitrogen elemental distribution maps of TPU/PCL fibers obtained by EDS analysis. (j–m) SEM images of TPU/PCL films. (n–q) Nitrogen elemental distribution maps of TPU/PCL films obtained by EDS analysis. (PCL contents: 20, 40, 60, and 80%.)

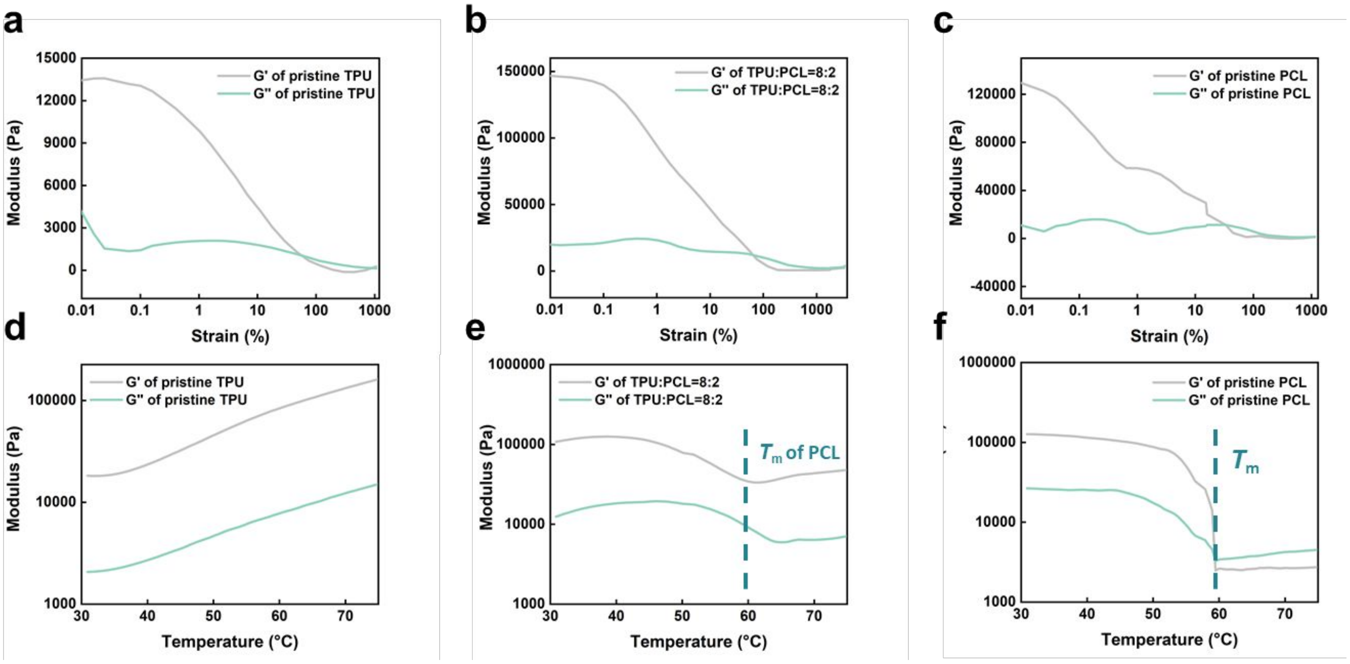

Figure S4. Rheological response of (a) TPU, (b) TPU/PCL, and (c) PCL measured in oscillatory amplitude sweep mode. Temperature-dependent rheological behavior of (d) TPU, (e) TPU/PCL, and (f) PCL fibers measured in heating mode.

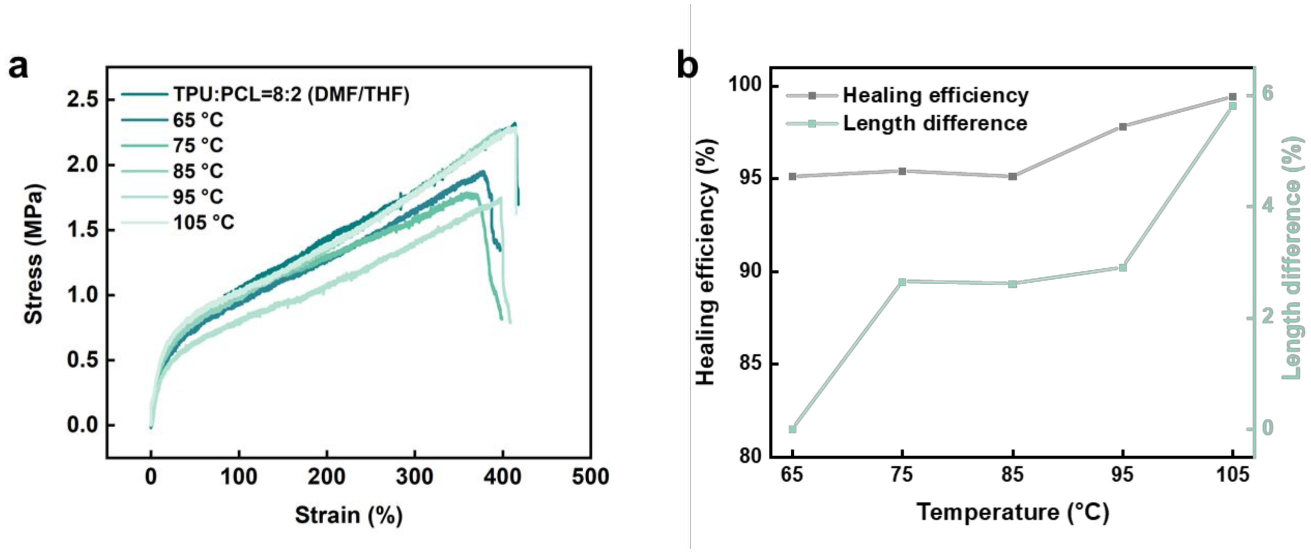

Figure S5. (a) Stress–strain curves comparing the mechanical response of healed fibers after healing at different temperatures. (b) Healing efficiency and fiber deformation as a function of healing temperature.
